# Encapsulation of Vitamin B_12_ by Complex Coacervation of Whey Protein Concentrate–Pectin; Optimization and Characterization

**DOI:** 10.3390/molecules27186130

**Published:** 2022-09-19

**Authors:** Neda Akbari, Elham Assadpour, Mohammad Saeed Kharazmi, Seid Mahdi Jafari

**Affiliations:** 1 Iran Dairy Industries Co., Golestan Pegah, Gorgan 49189-39911, Iran; 2 Food Industry Research Co., Gorgan 49189-39911, Iran; 3 Food and Bio-Nanotech International Research Center (Fabiano), Gorgan University of Agricultural Sciences and Natural Resources, Gorgan 49189-43464, Iran; 4 Faculty of Medicine, University of California, Riverside, CA 92679, USA; 5 Department of Food Materials and Process Design Engineering, Gorgan University of Agricultural Sciences and Natural Resources, Gorgan 49189-43464, Iran; 6 Nutrition and Bromatology Group, Department of Analytical Chemistry and Food Science, Faculty of Science, Universidade de Vigo, E-32004 Ourense, Spain; 7 College of Food Science and Technology, Hebei Agricultural University, Baoding 071001, China

**Keywords:** complex coacervation, encapsulation, pectin, WPC, vitamin B_12_

## Abstract

Vitamin B_12_ (VB_12_) is one of the essential vitamins for the body, which is sensitive to light, heat, oxidizing agents, and acidic and alkaline substances. Therefore, the encapsulation of VB_12_ can be one of the ways to protect it against processing and environmental conditions in food. In this work, the influence of pectin concentration (0.5–1% *w*/*v*), whey protein concentrate (WPC) level (4–8% *w*/*v*) and pH (3–9) on some properties of VB_12_-loaded pectin–WPC complex carriers was investigated by response surface methodology (RSM). The findings showed that under optimum conditions (1:6.47, pectin:WPC and pH = 6.6), the encapsulation efficiency (EE), stability, viscosity, particle size and solubility of complex carriers were 80.71%, 85.38%, 39.58 mPa·s, 7.07 µm and 65.86%, respectively. Additionally, the formation of complex coacervate was confirmed by Fourier-transform infrared (FTIR) spectroscopy and atomic force microscopy (AFM). In addition, it was revealed that the most important factor in VB_12_ encapsulation was pH; at a pH < isoelectric point of WPC (pH = 3), in comparison with higher pH values (6 and 9), a stronger complex was formed between pectin and WPC, which led to an increase in EE, lightness parameter, particle size and water activity, as well as a decrease in the zeta-potential and porosity of complex carriers.

## 1. Introduction

Vitamin B_12_ (VB_12_), as a water-soluble nutrient, is an essential vitamin meaning that it is not produced by the body and must be supplied by external sources such as food products [1]. It is important for brain function and the synthesis of red blood cells; its deficiency can lead to neurological difficulties and anemia [2]. Although VB_12_ is found naturally in animal products such as meat, fish, eggs and dairy products, the fortification of some plant products with it is recommended to maintain public health; however, there is a serious limitation due to the high sensitivity of VB_12_ to destructive factors and harsh environmental conditions such as heat and oxidants. To overcome this limitation, encapsulation is recommended to maintain the unique properties of VB_12_ against external factors [3,4,5]. 

There are various techniques for encapsulating vitamins including spray drying, spray chilling and spray cooling, emulsion technique, fluidized bed coating, liposome entrapment and coacervation) [3,6]. Coacervation is a simple and popularly method for the development of polysaccharide-based nanocapsules [7,8]. It is categorized into simple and complex coacervation. In simple coacervation, the water-soluble solvents are added and changes in pH are based on the type of electrolyte added, whereas in complex coacervation, the oppositely charged colloids lead to alterations in pH, which causes immediate desolvation [9,10]. Mohammadi et al. reported that olive leaf extract in WPC–pectin complexes was more stable than when the WPC complex was used alone [11]. In another study, similar results were obtained for WPC–pectin loading saffron extract [12]. Complex coacervation occurs between at least two biopolymers when they have different charges. In fact, there are two phases in this phenomenon: one phase rich in solvent with low biopolymer concentrations and one phase rich in biopolymers that is known as coacervate [13]. Proteins have different charges at higher (negative) and lower (positive) pH values than their isoelectric point (pI), and therefore can be electrostatically bonded to anionic polysaccharides such as pectin at pH < pI and cationic polysaccharides such as chitosan at pH > pI [14].

WPC is a by-product produced by ultrafiltration, diafiltration and thermal evaporation, containing proteins with a concentration of 20–80% [15]. WPC is a natural emulsifier and stabilizer with the ability to produce a stable kinetic system [16]. The pI of WPC is about five, which means that it can form coacervate complexes with anionic polysaccharides at pH values < pH 5. Pectin is an heteropolysaccharide composed of α-(1,4)-galacturonic acid (GalA) units esterified with natural sugars [17]. WPC is widely used as a thickener, texturizer and emulsifying agent in food and pharmaceutical industries due to its high water absorption capacity [18,19]. Pectin is also an anionic polysaccharide and therefore can form complex coacervates with different proteins such as WPC [14]. At pH < pI, proteins are positively charged and react with negatively charged anionic groups of polysaccharides. These reactions are influenced by many factors such as pH, ionic strength, biopolymer addition, charge density, and the concentration of proteins and polysaccharides [20,21]. 

The complex coacervation of WPC and pectin depends on different factors such as pectin concentration, WPC concentration and pH value. So far, no study has explored the use of WPC and pectin complexes to encapsulate VB_12_. Based on the research and articles published so far, there is no report on the coacervation of VB_12_ using WPC and pectin complexes. The use of these two edible and safe biopolymers is an innovation for the coacervation of VB_12_. Therefore, the present study aimed to optimize the conditions of VB_12_ encapsulation by the coacervate formation process between WPC and pectin. For this purpose, the variables of pectin concentration, WPC concentration and pH value were optimized using the statistical method of RSM. Additionally, the EE factor of VB_12_, stability, viscosity, particle size and solubility were examined as responses. After obtaining the most optimal operating conditions according to the obtained answers, FTIR and AFM analyses were performed on the optimal samples.

## 2. Results and Discussion

### 2.1. Statistical Analysis of the Responses and Fitted Models

As mentioned, in this study, an RSM design with CCRD (17 runs) (Table 2) including eight factorial points, six axial points and three center points was carried out to optimize the effect of independent variables, including pectin concentration, WPC concentration and pH, on the properties of complex carriers loaded with VB. As shown in Table 2, the lower and upper limits for different responses were: 39.5 and 99.5% for the EE related to run = 7 (pectin = 0.5%, WPC = 8% and pH = 9) and run = 4 (pectin = 1%, WPC = 8% and pH = 3), 60 and 98% for the stability related to runs of 1 and/or 13 (pectin = 0.5 and/or 0.75%, WPC = 4 and/or 6% and pH = 3) and run = 12 (pectin = 0.75%, WPC = 8% and pH = 6), 30.4 and 42.7 mPa·s for the viscosity related to runs 13 and 2 (pectin = 1%, WPC = 4% and pH = 3), 1 and 99.5 µm for the particle size related to runs 7 and 4, and 37 and 78% for solubility related to runs 2 and 8 (pectin = 1%, WPC = 8% and pH = 9), respectively.

The obtained data were subjected to ANOVA using Design expert software (version 11). The means were compared using the Duncan’s multiple ranges test at a significant level of *p* < 0.05 (Table 1). The high F-value (13.1, 5.81, 7.49, 74.06 and 3.89 for EE, stability, viscosity, particle size and solubility, respectively), low *p*-value (0.001, 0.015, 0.007, <0.001 and 0.043 for EE, stability, viscosity, particle size and solubility, respectively) and insignificant lack-of-fit for all suggested models showed that the obtained models were highly significant and the experimental data were well fitted with them [22]. In addition, the determination coefficient values (94.39, 88.19, 90.60, 98.96 and 83.34% for EE, stability, viscosity, particle size and solubility, respectively) indicated that only limited data were unpredictable with the suggested models, and there were excellent correlations between the experimental and predicted values [23]. The proposed models are shown below:

(1) $$\mathrm{EE}=85.41+0.71A+0.69B-25.4C+2.76\mathrm{AB}+1.01\mathrm{AC}-3.29\mathrm{BC}-0.55A^{2}-3.05B^{2}-11.8C^{2}\;$$

(2) $$\mathrm{Stability}=84.44+0.5A+2.2B+6.35C-0.75\mathrm{AB}+1.25\mathrm{AC}-1.38\mathrm{BC}-0.9A^{2}+4.1B^{2}-17.15C^{2}\;$$

(3) $$\mathrm{Viscosity}\;=36.14+3.23A-0.79B+0.67C-0.95\mathrm{AB}+0.3\mathrm{AC}+0.97\mathrm{BC}+0.28A^{2}+4.38B^{2}-3.52C^{2}\;$$

(4) $$\mathrm{Particle}\;\mathrm{size}=6.55+7.72A+4.24B-34.67C+3.37\mathrm{AB}-3.81\mathrm{AC}-2.11\mathrm{BC}-2.86A^{2}+8.56B^{2}+29.73C^{2}\;$$

(5) $$\mathrm{Solubility}=51.9+5.2A+0.64B+8.6C+4\mathrm{AB}+7.75\mathrm{AC}+2\mathrm{BC}+4.42A^{2}-7.58B^{2}+7.42C^{2}\;$$

in which A, B, and C, are the linear effects, A^2^, B^2^, and C^2^ are the effects of squares, and AC, BC, and AB are the interaction effects.

If the *p*-values for each variable are >0.05 (at the 5% level), it indicates that the variable is not significant in the model, and if it is less than this value it indicates that the variable is significant. According to Table 1, depicting results of the ANOVA of pectin concentration (A) and WPC concentration (B), both the linear and quadratic conditions as well as the interaction of AB, AC, and BA at the 5% level had no significant effect, but the other variable, the pH, in the linear and quadratic conditions had a significant effect on the EE, stability, and solubility of the samples. The variable of pectin concentration in the linear mode, as well as WPC concentration and pH in the quadratic conditions, had a significant effect, and the variables of WPC concentration and pH in the linear mode had no significant effect on viscosity. Based on results of particle size, all variables had a significant effect in the linear mode.

### 2.2. The Effect of Independent Variables on the Responses

**Figure 1:**
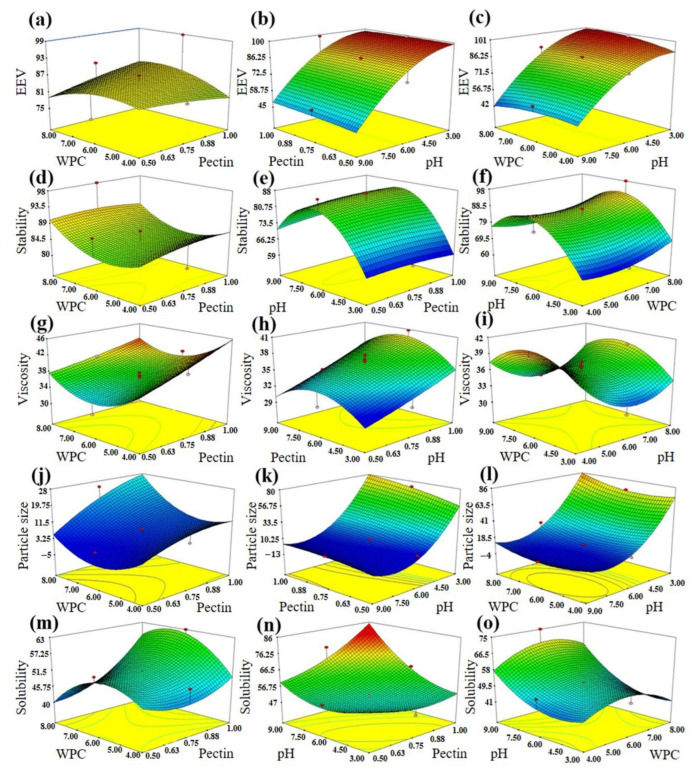
Effect of independent factors (pectin/WPC concentration and pH) on the encapsulation efficiency (EE) (**a**–**c**), stability (**d**–**f**), viscosity (**g**–**i**), particle size (**j**–**l**) and solubility (**m**–**o**) of vitamin B_12_–loaded complexes.

The 3D graphs related to the effect of independent variables on different responses are shown in Figure 1. The interaction of WPC concentration, pectin concentration and pH on EE is investigated in Figure 1a–c. According to the results, when the interaction of WPI and pectin concentration was investigated, it can be concluded that the concentration changes of these two parameters alone do not have a significant effect on EE. However, when the WPC and pectin concentration are considered constant, the amount of EE increases in proportion to the decrease in pH. It can be concluded that the effect of pH is greater than the WPC and pectin concentration on the EE variable. The previous studies showed that the pI of WPC is about five and it can be electrostatically bonded to anionic polysaccharides such as pectin at pH values < pI [24]. Therefore, it can be stated that an increase in EE with increasing wall materials and decreasing pH was probably due to creating a better protective layer with a higher and stronger entrapment of VB_12_ within complex particles [25]. The obtained results were in line with the reported data [14] for the encapsulation of rose essential oil into WPC–pectin complexes. 

According to Figure 1e,f and Equation (2), it is obvious that pH plays a key role in the stability of VB_12_-loaded pectin–WPC complexes. Figure 1d–f shows the effect of the interaction of WPC concentration, pectin concentration and pH on the stability of the formed complex. According to the results, when the interaction of the two parameters of WPC and pectin concentration was investigated, it can be concluded that the concentration changes of these two parameters alone do not have a significant effect on stability. However, when the WPC and pectin concentration are considered constant, the amount of stability increases in proportion to the increase in pH. It can be concluded that the effect of pH is greater than the WPC and pectin concentrations on the stability variable. Additionally, stability increases up to about pH 6, after which a sharp decline is observed. As stated earlier, although there is a negative repulsion force between pectin and WPC in pH values > 5, the formation of a complex between pectin and WPC at higher pH values (~6) is due to the presence of electrostatic forces between the negative groups of pectin and the positive groups remaining in the protein [26]. However, it was observed that the stability decreased with an excessive increase in pH (>6) which may be related to the reduction of complex formation between pectin and protein due to the presence of similar electric charges on their surface. On the other hand, at low pH values (pH = 3), there is a strong attraction between pectin and WPC, which results in larger complexes with lower stability [12]. Based on the statistical data, the highest stability was observed at pH = 6; on average, between 80–98% stability was established. In other words, there was a decrease of 2–20% in vitamin B_12_ levels. At pH = 3, about 60–68% stability was observed in different treatments, and a 32–40% decrease in vitamin B_12_ was observed. At pH = 9, a 72–80% stability was observed and 20–28% reduction in vitamin B_12_ was obtained.

According to the results obtained from previous investigations, pectin with DE < 50 has more GalA values than pectin with DE > 50. The DE and GalA values of the two pectins show that LMP has a significantly higher total charge density than HMP, because there is a greater amount of carboxylic acid groups in GalA residues in LMP that enhances ionic–dipole moments with the aqueous phase [27,28]. Lan et al. [19] tested the DM effect of pectin using two commercial samples of citrus pectin DM 81 and 35%, respectively. They found that LMP exhibits primary interaction at a lower pH than HMP due to its higher “total charge density”.

Figure 1g–i have evaluated the interaction of WPC concentration, pectin concentration and pH on the viscosity of the formed complex. According to the results, when the influence of the two parameters, WPC concentration and pectin concentration, was examined, it can be concluded that increasing the concentration of pectin has a significant effect on increasing viscosity, and increasing the concentration of WPC does not have a significant effect. Additionally, the highest viscosity has been reported at pH 6. Viscosity is one of the most important parameters in the production of different complexes between polysaccharides and protein. From Table 1, Equation (3), and Figure 1g–i, it can be inferred that pectin concentration was the most important factor influencing viscosity, and there is a direct relationship between this factor and viscosity because pectin has a hydrophilic nature with a high capacity to absorb water that can increase the viscosity of different solutions [18]. Zeeb et al. [29] tested the effect of apple pectin concentration on pea and potato protein. They stated that in low and medium concentrations, neutral protein–pectin complexes are formed and precipitate. Above the critical concentration of pectin, the complexes were negatively charged and soluble, and an increase in viscosity was also observed. On the other hand, as can be seen in Figure 1h,i, with an increase in pH, viscosity was increased and then decreased. Generally, the increase in viscosity at a higher pH up to ~6 could be attributed to the higher stability of complexes in the mentioned pH value. In addition, a decrease in viscosity with a increasing pH from six to nine was probably due to the reduction of complex formation between pectin and protein, resulting in a reduction of loaded VB and water trapped in the capsules.

**Figure 2:**
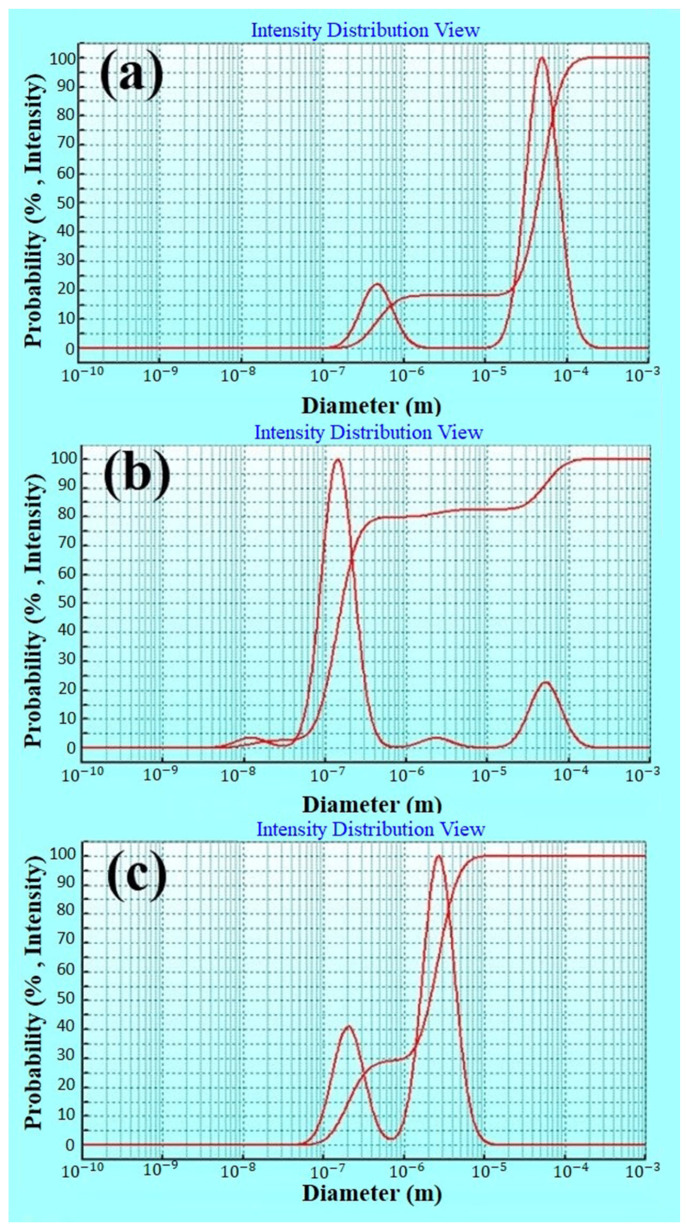
Particle size distribution of complex carriers containing vitamin B_12_ at (**a**) pH = 3, (**b**) pH = 6, and (**c**) pH = 9.

According to obtained results (Figure 1), when the concentration of WPC is considered constant, the size of particles increases in proportion to higher level of pectin. On the contrary, when the pectin concentration is considered constant in the graph, the particle size increases slowly with increasing WPC concentration. It can be concluded that the effect of pectin concentration is greater than WPC concentration. Additionally, in the comparison between pectin concentration and WPC concentration with pH, pH has a greater effect on particle size. The DLS results (more details in the following sections) indicated that complexes formed at pH > pI had a significant negative charge with smaller sizes (Figure 2) that can prevent the precipitation of complexes by repulsion forces and thereby increase stability [30].

Figure 1m–o show the interaction of WPC concentration, pectin concentration and pH on the solubility of the formed complex. From the interactive effect of WPC concentration and pectin concentration, it can be concluded that increasing the concentration of pectin has a significant effect on increasing solubility. Additionally, the figures show that increasing the concentration of WPC has no significant effect on solubility and, Figure 1n shows that increasing the concentration of pectin and pH increased the solubility of the complex. One of the most important parameters in the food and pharmaceutical applications of encapsulated bioactive compounds is their solubility. Table 1, Equation (4), and Figure 1m–o illustrate that pH value was the most important factor in the determination of solubility; this parameter increased with raising the pH. It can be stated that at pH = 3, in comparison with higher pH values, the formed complexes between pectin and WPC are stronger, which can lead to a decrease in solubility, while the complexes formed in higher pH values such as pH = 9 are weaker and the probability of separating their wall materials is more and thereby their solubility is higher [8,27,31]. Soluble one-phase complexes of proteins and polysaccharides are formed when the electrostatic interactions are fairly weak and the net charge is relatively high. Complex coacervations are formed when the electrostatic interactions are stronger and the net charge is low [30]. Giancone et al. [32] evaluated the interaction of soy flour with soy at pH 4.6 below the pI of soy protein in order to prevent unstable insoluble protein complexes during film formation. Probably due to the formation of electrostatic complex with pectin, the turbidity of the protein solution decreased and the solubility increased. In addition, DLS data (Figure 2) indicated that the size of microcapsules created at pH = 3 was larger than those formed at pH = 9, and therefore it takes longer to solve them [33].

Based on RSM results, the variable pH has a significant effect on the particle size. In order to further discuss this, the DLS diagram of the samples prepared at three different pHs (3, 6 and 9) was examined. Figure 2 shows that the smallest particles size was related to the complexes formed at pH = 6, which had the highest stability. The obtained results were in agreement with the reported data for folic acid encapsulated into maltodextrin–whey protein double emulsions and sodium ascorbate loaded into pectin–whey protein isolate complexes, respectively [30,34]. As shown in Figure 2, the particle size of complex carriers increased at lower pH values which can be possibly attributed to the formation of a strong complex of coacervate and attractive interactions between the two oppositely charged biopolymers; this can lead to trapping water, reducing the vitamin release and thereby increasing particle size [14,18,35]. 

### 2.3. Optimization of Conditions for Loading VB_12_ into Pectin–WPC Complex Carriers 

Based on the previous results and discussion, an optimization study is required to find the best conditions for encapsulation process. As mentioned before, independent variables were pectin/WPC concentration and pH, while the responses were the EE (maximize with importance of five), stability (in range), viscosity (in range), particle size (minimize with importance of three) and solubility (maximize with importance of four) of the formed microcapsules. The optimized conditions were obtained at pectin = 1% *w*/*v*, WPC = 6.47% *w*/*v* and pH = 6.6; in these conditions, EE, stability, viscosity, particle size and solubility were predicted to be 80.71%, 85.38%, 39.58 mPa·s, 7.07 µm and 65.86%, respectively. In order to ensure the accuracy of the predicted models, three experiments were performed in the mentioned optimum conditions and the obtained results (78.27 ± 1.08%, 85.46 ± 0.87%, 38.21 ± 1.22 mPa.s, 6.14 ± 0.17 µm and 65.12 ± 1.14% for EE, stability, viscosity, particle size and solubility, respectively) were very close to the predicted ones, which confirmed them. 

Based on the results obtained from the optimization, the ratio of pectin concentration to WPC concentration is 1 to 6.47 (wt%) [19]; that is the optimal and suitable concentration for optimal coacervation and the encapsulation of VB_12_ [31,36] reported that the ratio of 1 to 6 (wt%) of pectin to protein (whey protein isolate) is proper for complex formation and in this condition the obtained complex is stable and has a suitable viscosity. Complexes are formed in a certain ratio of protein and polysaccharide under specific conditions of pH and ionic strength. When the concentration of a biopolymer increases due to the presence of neutral charges, soluble complexes are created. When the concentration of one biopolymer is high, the complex is not formed, because it increases the ions with the opposite charge in the solution, which increases the solubility of the complex by screening the charges of the biopolymer, preventing the formation of the complex [37].

### 2.4. Color of Complex Carriers

The color parameters are very important for sensory evaluation of food and drug products, including the encapsulated materials. The L* index indicates the lightness and should be as high as possible for clear products containing the encapsulated vitamins [38]. In this part, the effect of pH as the most important factor in complex formation on lightness of complex carriers was evaluated. For this purpose, the pH was considered variable from three to nine and other factors were constant at the optimum point (pectin = 1% *w*/*v* and WPC = 6.47% *w*/*v*). The findings depicted that the L* value for carriers formed at pH = 3, 6 and 9 was 73.54, 49.27 and 28.65, respectively. The previous studies showed that there is a direct relationship between EE and the lightness parameter. When a color compound such as VB with a reddish-pink color is encapsulated, the amount of color in the solution is reduced and the amount of brightness is increased [39]. The obtained results also agreed with the reported data for pectin–WPC complexes encapsulating *d*-limonene [39]. In addition, Rocha et al. reported that the lightness parameter for cooked cake containing the encapsulated lycopene was higher than that containing the free lycopene. These researchers claimed that encapsulation leads to an increase in the L* value of the food product containing lycopene, which is in line with the obtained data in our study [40]. 

### 2.5. Surface Charge of Complex Carriers

In this study, a ZP measurement was done for the characterization of the density of surface charge and to investigate the stability of electrostatic colloidal dispersion of pectin–WPC microcapsules loading VB_12_. To prepare the examined complexes, the optimized amounts of pectin and WPC obtained through the optimization method were used at pHs of 3, 6, and 9. It was revealed that the microcapsules produced at pH = 3, 6 and 9 had a ZP = +0.8, −1.6 and −8.7 mV, respectively. At pHs < pI of WPC (pH = 3), pectin and WPC have opposite charges and a strong tendency to form complex coacervates. Thereby the positive charges on WPC are neutralized with the negative charges on pectin. The interaction of positive and negative charges cause ZP to be close to zero at pH = 3. With the increase of pH > pI of WPC, the amount of surface OH of amino acids becomes more than the amount of surface H, so the charge of the protein becomes negative, and pectin also has a negative charge due to the presence of carboxyl groups on its surface. 

Therefore, at a high pH, the repulsive forces between whey protein and pectin are stronger than the electrostatic attraction forces between the anionic carboxyl groups of pectin and the positively charged subunits of whey protein, although in this condition, there is an electrostatic reaction between the negative charges of the polysaccharide and the positive charges of the protein, and this causes the formation of a complex. Additionally, the ZP of the complex formed at pH > pI of WPC increases (ZP becomes negative) and prevents the precipitation of the complex. Therefore, the complexes remain suspended and dispersed in the suspension and create a more stable complex. In general, they may result from a strong attraction between a positively charged protein and a negatively charged polysaccharide at pH < pI of the protein, from weak attractive interactions between uncharged or negatively charged proteins and positively charged polysaccharides at pH > pI, as well as from the formation of local complexes between positively charged regions of the protein molecules and a negatively charged polysaccharides at pH > pI [41,42]. The findings are in agreement with the reported results by Peinado et al. [43] and Lutz et al. [44] for heat-treated lactoferrin–anionic polysaccharides and modified pectin–whey protein isolate complexes, respectively. Additionally, our results are in accordance with a previous reported study which explored the application of gelatin, alginate and pectin coacervation [45].

### 2.6. Water Activity and Porosity of Complex Carriers

The a_w_ and porosity of microcapsules prepared at different pH values were determined, while pectin and WPC concentrations were considered to be constant at the optimum point. The a_w_ of complex carriers in pH = 3, 6 and 9 was 0.178, 0.092 and 0.075, while the porosity at these pH values was 0.70, 0.74 and 0.78, respectively. As can be seen, there is an inverse relationship between a_w_ and porosity, and with an increase in pH value, a_w_ was decreased whereas the porosity was increased. As mentioned earlier, a strong complex was formed between pectin and WPC at pH = 3, which led to the entrapment of VB_12_ together with water that increased its density, as well as increased aw and decreased porosity. These changes did not occur at pH = 9 due to weaker electrostatic forces between the wall materials [14]. Ghasemi et al. [39] studied pectin-WPC nanocomplexes for loading orange peel oil and reported similar results. These researchers mentioned that with an increase in pH from 3 to 9, a_w_ was decreased from 0.125 to 0.041 and the porosity was increased from 0.72 to 0.79, which shows a similar trend to the present study.

### 2.7. Chemical Interactions and Topology of Complex Carriers

**Figure 3:**
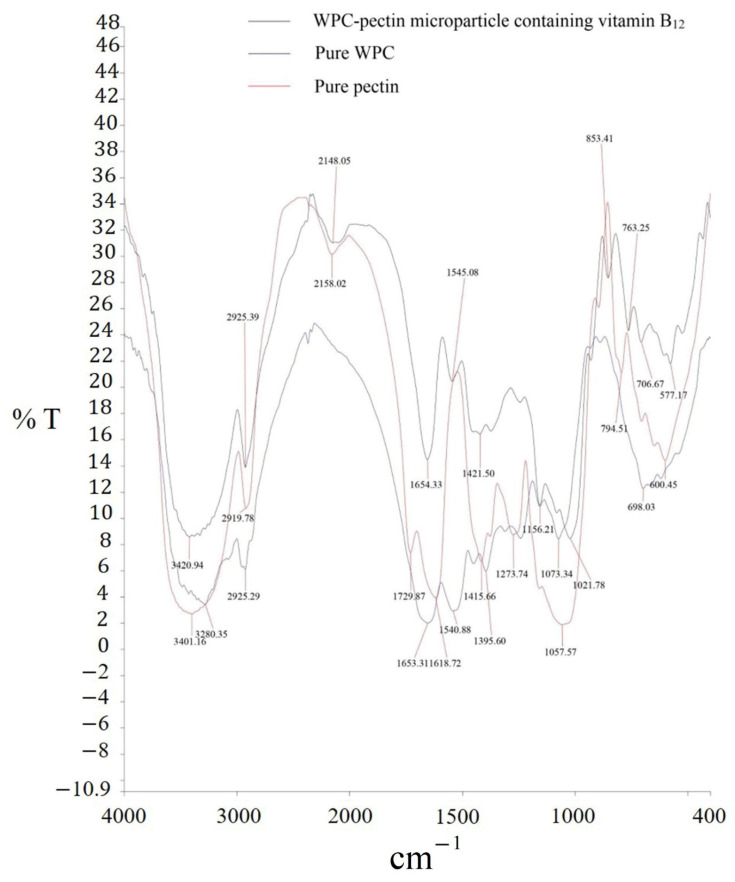
FTIR spectra of pure pectin, WPC and WPC–pectin complex carriers containing vitamin B_12_.

In this step, the FTIR spectra of pure pectin, WPC and pectin-WPC complexes loaded with VB and obtained under optimum conditions were analyzed. The formation of the amide groups is predictable in the event of the reaction between the carboxyl groups of pectin and the amino groups of WPC [34]. The FTIR spectrum of pure pectin is shown in Figure 3. The characteristic peaks of 3300–3500, 2919.78, 1729.87, 1618.72, 1415.66, 1273.74 and 1057.57 cm^−1^ were attributed to the hydrogen bonds of OH groups, C–H stretching vibrations of CH, CH_2_ and CH_3_, esterified carboxyl groups (COOCH_3_) of GalA, free carboxylic groups (–COO^−^), C–H bending vibrations, C–O–C vibrations of glycosides and the presence of pyranose and furanose in pectin, respectively [22,46]. In the FTIR spectrum of native WPC, the signals that appeared in 3280.35, 2925.29, 1653.31, 1540.88 and 1073.34 cm^−1^ were related to the groups of O–H, C–H, C–O, N–H and C–N in the WPC structure [39,47]. On the other hand, in the spectrum of pectin–WPC complexes loaded with VB, it can be seen that a new sharp signal appeared at 1654.33 cm^−1^, belonging to amide bonds between pectin and WPC, which confirms the complex coacervate formation between these two biopolymers [27,48,49]. 

**Figure 4:**
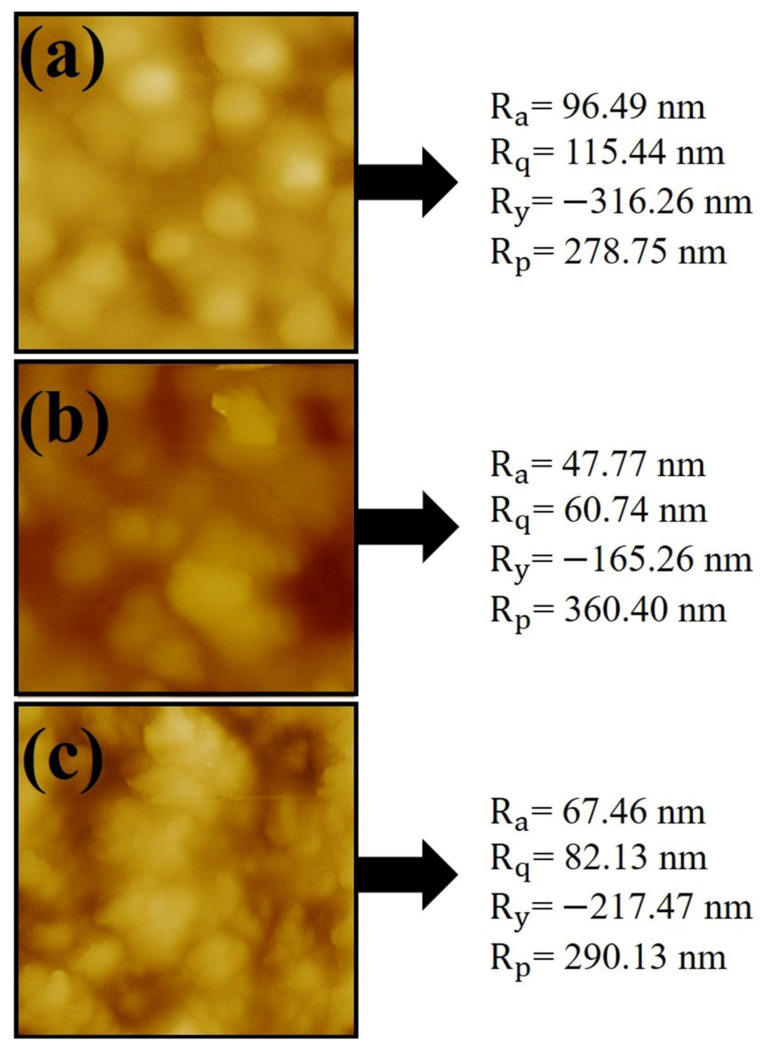
AFM images of complex carriers containing vitamin B_12_ at (**a**) pH = 3, (**b**) pH = 6, and (**c**) pH = 9.

The surface topography of the microcapsules prepared in pH values of 3, 6 and 9 (pectin = 1% and WPC = 6.47% were constant at optimum point) was evaluated and the obtained results are shown in Figure 4. As can be seen, pectin–WPC complexes encapsulating VB and prepared at all pH values were in the spherical shape with a micro size. In addition, it is visible that the complexes created at pH = 3 were more spherical and larger than those created in other pH values. These results can be attributed to the formation of the strong pectin–WPC complexes containing VB at pH = 3 and confirms the obtained results from DLS data [14,39].

## 3. Materials and Methods

### 3.1. Materials

WPC (protein = 80%, ash = 3.5%, moisture = 5%) was obtained from Lactoprot Deutschland GmbH (Germany). Pectin (LMP, (DE < 50%, LM–12 CG, Lot # GR74485, Mw = 76 kDa, 65% of GalA, as reported by the manufacturer) and vitamin B_12_ (purity ≥ 98%) were provided from Sigma Chemical Co. (St. Louis, MO, USA). All other chemicals and reagents were of analytical grade.

### 3.2. Optimization of WPC–Pectin Complexation

The three variables of pectin concentration, WPC concentration, and pH were optimized using the RSM. The response level method is usually used to investigate the combined effect of several variables in order to find the optimal conditions for a multivariate condition. The encapsulation efficiency (EE) of VB_12_, as well as its stability, viscosity, particle size, and solubility of formed complexes were measured as dependent variables (responses). The WPC level (4–8% *w*/*v*), pectin level (% *w*/*v*), and pH (3–9) as independent variables were selected (Table 2). A central composite rotatable design (CCRD) with 17 runs was conducted to evaluate the variables (Table 3). With CCRD, three levels for each factor are used, which enables one to fit second-order polynomials to the experimental data points. Both linear and quadratic effects of the three variables under study, as well as their interactions, on the dependent variable were calculated. Their significance was evaluated by an analysis of variance (ANOVA). The experimental design results were fitted by a second-order model equation in order to correlate the response to the independent variables.

**Table 2:** Independent variables and their levels used in the central composite rotatable design.

Factors	Coded Symbols	Levels		
		−1	0	1
Pectin concentration (% *w*/*v*)	A	0.5	0.75	1
WPC concentration (% *w*/*v*)	B	4	6	8
pH	C	3	6	9

**Table 3:** Central composite rotatable design with experimental values of responses based on independent variables.

Run	Independent Variables			Measured Responses				
	Pectin (%)	WPC (%)	pH	EE (%)	Stability (%)	Viscosity (mPa·s)	Particle Size (µm)	Solubility (%)
1	0.5	4	3	99.2	60	35.1	63.23	61
2	1	4	3	85.3	65	42.7	79.19	37
3	0.5	8	3	97.8	68	34	63.57	41
4	1	8	3	99.5	64.5	36.2	99.5	55
5	0.5	4	9	49.5	75	34.3	2.6	45
6	1	4	9	44.2	79.5	41.5	9.84	74
7	0.5	8	9	39.5	72	35.5	1	55
8	1	8	9	40.7	79	40.5	15.2	78
9	0.5	6	6	75.4	88	30.5	2.64	53
10	1	6	6	98.8	80	40.8	6.51	63
11	0.75	4	6	80.8	80	40	7	49
12	0.75	8	6	88.4	98	39.5	25	43
13	0.75	6	3	98.9	60	30.4	72.1	47
14	0.75	6	9	52.8	75.5	33.3	2.24	75
15	0.75	6	6	80.1	82	37	4.3	51
16	0.75	6	6	80.2	82.5	36.7	3.4	51
17	0.75	6	6	87	87	37.8	8.41	47

#### 3.2.1. Preparation of Biopolymer Stock Solutions

Preparation of the biopolymeric solutions (4–8% *w*/*v*) was done in such a way that different levels of WPC were dissolved in 100 mL distilled water and were refrigerated overnight. In the same way, pectin solutions were prepared by dissolving pectin powder (0.5–1 g/100 mL) in distilled water under stirring (70 °C, 30 min) and keeping it at room temperature for complete rehydration [26].

#### 3.2.2. Preparation of WPC–Pectin Complex Carriers for Loading VB_12_

Biopolymer stock solutions with a specified volume and concentration were first prepared and mixed at room temperature for 10 min by a magnetic stirrer. In the next step, the obtained mixtures were homogenized by an ultrasonic device (20 kHz, Ultrasonic Technology Company, Tehran, Iran) for 10 min at 25 °C and 350 W. During homogenization, VB_12_ was added gradually at a concentration of 20% *w*/*w* of the total solids. To form complex carriers, the pH of the obtained solutions was adjusted at 3–9 by 1 N HCl and NaOH according to Table 2. Finally, the complexes were freeze-dried for 48 h to obtain encapsulated powders [50].

### 3.3. Encapsulation Efficiency of VB_12_

EE was determined using spectroscopy method described with some modifications [39]. For this purpose, two parameters of the total content (TC) and surface content (SC) of VB in the obtained powders were measured. To determine the total content of VB, powders (0.5 g) were dissolved in 20 mL of deionized water on a magnetic stirrer for 10 min and then heated in a water bath (50 °C, 15 min) to completely destroy the complexes. Afterwards, ethanol (20 mL) was added and the obtained mixture was vortexed (5 min) and centrifuged at 3500 rpm for 20 min (Centrifuge Universal 320, Hettich, Germany). Eventually, the absorbance of the sample after dilution with deionized water was read at 550 nm and the VB_12_ content was determined by comparing the obtained absorbance with the standard curve of VB (0.01–0.6 mg/mL). To determine the surface content of VB_12_, 0.5 g of the freeze-dried powder was dissolved in 20 mL ethanol, and was then vortexed (2 min) and filtered (Whatman paper no. 41), respectively. Then, the absorbance of sample after dilution with deionized water was read at 550 nm and the content of VB was determined using the standard curve. Finally, EE was calculated as follows:

(6) $$\mathrm{EE}\;\left(\%\right)=\frac{\mathrm{TC}-\mathrm{SC}}{\mathrm{TC}}\times 100\;$$

### 3.4. Stability Analysis of Complex Carrier Solutions

For this purpose, 10 mL of complex solutions was centrifuged (20,000× *g*, 30 min, 25 °C) and the sediment volume was determined by a graduated cylinder. The stability of the formed complexes was calculated by the following equation [50]:

(7) $$\mathrm{Stability}\;\left(\%\right)=\frac{\mathrm{The}\;\mathrm{volume}\;\mathrm{of}\;\mathrm{sediment}}{\mathrm{The}\;\mathrm{volume}\;\mathrm{of}\;\mathrm{primary}\;\mathrm{complexes}}\times 100\;$$

### 3.5. Viscosity Analysis of Complex Carrier Solutions

To determine the viscosity, 16 mL of complex solution was poured into the viscometer cylinder (Myr 3000 V2R, Viscotech, El Vendrell, Spain) and the viscosity was measured using spindle L3 at a temperature and shear rate of 25 °C and 18.3 s^−1^, respectively.

### 3.6. Particle Size Measurement of Complex Carrier Solution

The average particles size was determined by the dynamic light-scattering (DLS) method (Zetasizer, Malvern, UK). For this purpose, the samples were diluted with distilled water and the average particle size was measured.

### 3.7. Solubility Analysis of Encapsulated Powders

For this purpose, an aqueous solution of powdered sample (1% *w*/*v*) was first produced and then the obtained mixture was centrifuged at 3000× *g* for 5 min. In the next step, the formed sediment was dried on a Petri dish at 105 °C for 5 h. The solubility was calculated as follows:

(8) $$\mathrm{Solubility}\;\left(\%\right)=\frac{W_{1}-W_{2}}{W_{1}}\times 100\;$$

where, W_1_ is the weight (g) of initial powder and W_2_ is the weight (g) of dried sediment.

### 3.8. Analysis of Optimum Encapsulated Samples

#### 3.8.1. Analysis Color of Complex Carrier Solution

The lightness parameter (L*) was evaluated by image analysis using Image J software.

#### 3.8.2. Water Activity

Water activity (a_w_) was determined using a water activity meter (Novasina LabMaster, UK).

#### 3.8.3. Porosity and Density

The porosity was calculated according to the equation below:

(9) $$\varepsilon =1-\left(\frac{\rho_{b}}{\rho_{a}}\right)\;$$

where, ε is porosity, $\rho_{b}$ is bulk density and $\rho_{a}$ is absolute density.

The bulk density was calculated as follows:

(10) $$\rho_{b=}\frac{m_{p}}{V_{p}}\;$$

where, $m_{p}$ and $V_{p}$ are the weight and volume of 2 g powdered samples, respectively. Additionally, to determine the absolute density, the toluene displacement method by a pycnometer was applied according to the following equation [26]:

(11) $$\rho_{a}=\frac{m_{ps}-m_{p}}{V_{t}}\;$$

where, $m_{ps}$ is the weight of (pycnometer + powder sample), $m_{p}$ is the weight of pycnometer and $V_{t}$ is the volume of toluene. The volume of toluene ($V_{t}$) was calculated based on:

(12) $$V_{t}=\frac{\left(m_{pt}-m_{p}\right)-\left(m_{pst}-m_{ps}\right)}{\rho_{t}}\;$$

where, $m_{pt}$ is the weight of (pycnometer + toluene), $m_{pst}$ is the weight of (pycnometer powder sample + toluene) and $\rho_{t}$ s toluene’s density.

#### 3.8.4. Zeta Potential (ZP)

The ZP of the complexes formed based on the optimization formulation was determined against the pH change at 45 °C. The pH of the specific dispersion was adjusted by the addition of acetic acid (1%, *v*/*v*) and NaOH 1 M. To prevent multiple scattering effects, the samples were diluted with a buffer of the same pH value prior to analysis so that the droplet concentration was approximately 0.05 g/100 mL. The ZP values were determined by a Zetasizer Nano-ZS (Malvern, UK). 

#### 3.8.5. FTIR Spectroscopy

FTIR spectroscopy of the obtained complex powder samples was done by an FTIR spectrometer (Spectrum RX1, PerkinElmer, Waltham, MA, USA) using the KBr disk method. It should also be noted that the wavenumber was ranged from 400 to 4000 cm^−1^ with a resolution of 4 cm^−1^.

#### 3.8.6. Morphology of Powders

An atomic force microscope (AFM; DualScopeTM DS95-50, Freital, Germany) was applied to evaluate the surface topography of complex powder samples.

### 3.9. Statistical Methods

In the current study, RSM and CCRD with three variables at three levels were applied to evaluate the effect of independent variables on the five responses of EE, stability, viscosity, particle size and solubility of the formed complexes in quadratic models. All the computation and graphic generation were performed using the statistical software Design Expert (v.8, Minneapolis, Min, USA). All measurements were repeated at least twice using freshly prepared samples and data are expressed as means ± SD.

## 4. Conclusions

In the current study, a central composite design with three variables (pectin/WPC concentration and pH) in three levels was used to optimize the formation conditions of pectin–WPC complexes for loading vitamin B_12_. The obtained results showed that EE, stability, viscosity, particle size and solubility were 80.71%, 85.38%, 39.58 mPa·s, 7.07 µm and 65.86% under optimum encapsulation conditions (pectin = 1% *w*/*v*, WPC = 6.47% *w*/*v* and pH = 6.6), respectively. FTIR analysis indicated that amide bonds were formed between pectin and WPC, confirming the complex coacervate production by these two wall materials. In addition, the statistical analysis suggested that the pH value was the most important factor in the encapsulation process; with a decrease in this factor, EE, the lightness parameter, particle size and a_w_ of complexes were increased and ZP and porosity of them were reduced. Also, DLS and AFM analyses proved that the formed complexes were of a micro size. Generally, it is recommended that the method used in this work can be considered as a high potential encapsulation technique for water-soluble vitamins aimed at food and drug applications.

## Figures and Tables

**Table 1:** The results of ANOVA for properties of complex carriers loaded with vitamin B_12_.

Source	EE		Stability		Viscosity		Particle Size		Solubility	
	F-Value	*p*-Value	F-Value	*p*-Value	F-Value	*p*-Value	F-Value	*p*-Value	F-Value	*p*-Value
Model	13.1	0.001	5.81	0.015	7.49	0.007	74.06	0.000	3.89	0.043
A-pectin	0.08	0.785	0.09	0.778	35.77	0.000	22.53	0.002	4.84	0.063
B-WPC	0.08	0.791	1.66	0.238	2.14	0.187	6.80	0.035	0.06	0.807
C-pH	102.5	0.000	13.8	0.007	1.54	0.254	454.5	0.000	13.23	0.008
AB	0.97	0.357	0.15	0.706	2.48	0.159	3.43	0.106	2.29	0.174
AC	0.13	0.729	0.43	0.533	0.25	0.634	4.38	0.074	8.60	0.022
BC	1.37	0.280	0.52	0.494	2.61	0.150	1.35	0.283	0.57	0.474
A^2^	0.01	0.913	0.08	0.792	0.007	0.795	0.83	0.392	0.94	0.365
B^2^	0.4	0.549	1.55	0.253	17.63	0.004	7.43	0.029	2.75	0.141
C^2^	5.92	0.045	27.06	0.001	11.38	0.011	89.55	0.000	2.64	0.148
Residual										
Lack-of-Fit	5.23	0.168	4.98	0.176	12.23	0.077	4.79	0.182	14.27	0.067
R^2^	0.9439		0.8819		0.9060		0.9896		0.8334	
Adj R^2^	0.8718		0.7300		0.7850		0.9762		0.6191	

## Data Availability

Not applicable.

## References

[1] Watanabe F. (2007). Vitamin B12 sources and bioavailability. Exp. Biol. Med..

[2] Watanabe F., Takenaka S., Kittaka-Katsura H., Ebara S., Miyamoto E. (2002). Characterization and bioavailability of vitamin B12-compounds from edible algae. J. Nutr. Sci. Vitaminol..

[3] Katouzian I., Jafari S.M. (2016). Nano-encapsulation as a promising approach for targeted delivery and controlled release of vitamins. Trends Food Sci. Technol..

[4] Sanguansri P., Augustin M.A. (2006). Nanoscale materials development—A food industry perspective. Trends Food Sci. Technol..

[5] Siddiq M., Pascall M.A. (2018). Peas, sweet corn, and green beans. Handbook of Vegetables and Vegetable Processing.

[6] Schmitt C., Turgeon S.L. (2011). Protein/polysaccharide complexes and coacervates in food systems. Adv. Colloid Interface Sci..

[7] Ezhilarasi P., Karthik P., Chhanwal N., Anandharamakrishnan C. (2013). Nanoencapsulation techniques for food bioactive components: A review. Food Bioprocess Technol..

[8] Moschakis T., Biliaderis C.G. (2017). Biopolymer-based coacervates: Structures, functionality and applications in food products. Curr. Opin. Colloid Interface Sci..

[9] Amine C., Boire A., Kermarrec A., Renard D. (2019). Associative properties of rapeseed napin and pectin: Competition between liquid-liquid and liquid-solid phase separation. Food Hydrocoll..

[10] Choi Y.-R., Chang Y.H. (2018). Microencapsulation of gallic acid through the complex of whey protein concentrate-pectic polysaccharide extracted from Ulmus davidiana. Food Hydrocoll..

[11] Mohammadi A., Jafari S.M., Assadpour E., Esfanjani A.F. (2016). Nano-encapsulation of olive leaf phenolic compounds through WPC–pectin complexes and evaluating their release rate. Int. J. Biol. Macromol..

[12] Esfanjani A.F., Jafari S.M., Assadpour E. (2017). Preparation of a multiple emulsion based on pectin-whey protein complex for encapsulation of saffron extract nanodroplets. Food Chem..

[13] Veis A. (2011). A review of the early development of the thermodynamics of the complex coacervation phase separation. Adv. Colloid Interface Sci..

[14] Heydari M.K., Assadpour E., Jafari S.M., Javadian H. (2021). Encapsulation of rose essential oil using whey protein concentrate-pectin nanocomplexes: Optimization of the effective parameters. Food Chem..

[15] Albano K.M., Cavallieri Â.L.F., Nicoletti V.R. (2019). Electrostatic interaction between proteins and polysaccharides: Physicochemical aspects and applications in emulsion stabilization. Food Rev. Int..

[16] Dickinson E. (2008). Interfacial structure and stability of food emulsions as affected by protein–polysaccharide interactions. Soft Matter.

[17] Asgari K., Labbafi M., Khodaiyan F., Kazemi M., Hosseini S.S. (2020). Valorization of walnut processing waste as a novel resource: Production and characterization of pectin. J. Food Processing Preserv..

[18] Kazemi M., Amiri Samani S., Ezzati S., Khodaiyan F., Hosseini S.S., Jafari M. (2021). High-quality pectin from cantaloupe waste: Eco-friendly extraction process, optimization, characterization and bioactivity measurements. J. Sci. Food Agric..

[19] Lan Y., Ohm J.-B., Chen B., Rao J. (2020). Phase behavior, thermodynamic and microstructure of concentrated pea protein isolate-pectin mixture: Effect of pH, biopolymer ratio and pectin charge density. Food Hydrocoll..

[20] Jain A., Thakur D., Ghoshal G., Katare O., Singh B., Shivhare U. (2016). Formation and functional attributes of electrostatic complexes involving casein and anionic polysaccharides: An approach to enhance oral absorption of lycopene in rats in vivo. Int. J. Biol. Macromol..

[21] Ye A. (2008). Complexation between milk proteins and polysaccharides via electrostatic interaction: Principles and applications—A review. Int. J. Food Sci. Technol..

[22] Pasandide B., Khodaiyan F., Mousavi Z., Hosseini S.S. (2018). Pectin extraction from citron peel: Optimization by Box–Behnken response surface design. Food Sci. Biotechnol..

[23] Omar-Aziz M., Yarmand M.S., Khodaiyan F., Mousavi M., Gharaghani M., Kennedy J.F., Hosseini S.S. (2020). Chemical modification of pullulan exopolysaccharide by octenyl succinic anhydride: Optimization, physicochemical, structural and functional properties. Int. J. Biol. Macromol..

[24] Khoshmanzar M., Ghanbarzadeh B., Hamishekar H., Sowti M., Rezayi Mokarram R. (2013). Investigation of effective parameters on particle size, zeta potential and steady rheological properties of colloidal system based on carrageenan-caseinate nanoparticles. Res. Innov. Food Sci. Technol..

[25] Maleki O., Khaledabad M.A., Amiri S., Asl A.K., Makouie S. (2020). Microencapsulation of Lactobacillus rhamnosus ATCC 7469 in whey protein isolate-crystalline nanocellulose-inulin composite enhanced gastrointestinal survivability. LWT.

[26] Ghasemi S., Jafari S.M., Assadpour E., Khomeiri M. (2018). Nanoencapsulation of d-limonene within nanocarriers produced by pectin-whey protein complexes. Food Hydrocoll..

[27] Lan Y., Xu M., Ohm J.-B., Chen B., Rao J. (2019). Solid dispersion-based spray-drying improves solubility and mitigates beany flavour of pea protein isolate. Food Chem..

[28] Pillai P.K., Stone A.K., Guo Q., Guo Q., Wang Q., Nickerson M.T. (2019). Effect of alkaline de-esterified pectin on the complex coacervation with pea protein isolate under different mixing conditions. Food Chem..

[29] Zeeb B., Yavuz-Düzgun M., Dreher J., Evert J., Stressler T., Fischer L., Özcelik B., Weiss J. (2018). Modulation of the bitterness of pea and potato proteins by a complex coacervation method. Food Funct..

[30] Doublier J.-L., Garnier C., Renard D., Sanchez C. (2000). Protein–polysaccharide interactions. Curr. Opin. Colloid Interface Sci..

[31] Lan Y., Chen B., Rao J. (2018). Pea protein isolate–high methoxyl pectin soluble complexes for improving pea prtein functionality: Effect of pH, biopolymer ratio and concentrations. Food Hydrocoll..

[32] Giancone T., Torrieri E., Masi P., Michon C. (2009). Protein–polysaccharide interactions: Phase behaviour of pectin–soy flour mixture. Food Hydrocoll..

[33] Gharehbeglou P., Jafari S.M., Hamishekar H., Homayouni A., Mirzaei H. (2019). Pectin-whey protein complexes vs. small molecule surfactants for stabilization of double nano-emulsions as novel bioactive delivery systems. J. Food Eng..

[34] Assadpour E., Maghsoudlou Y., Jafari S.-M., Ghorbani M., Aalami M. (2016). Optimization of folic acid nano-emulsification and encapsulation by maltodextrin-whey protein double emulsions. Int. J. Biol. Macromol..

[35] Yuan Y., Wan Z.-L., Yang X.-Q., Yin S.-W. (2014). Associative interactions between chitosan and soy protein fractions: Effects of pH, mixing ratio, heat treatment and ionic strength. Food Res. Int..

[36] Chen B., Li H., Ding Y., Suo H. (2012). Formation and microstructural characterization of whey protein isolate/beet pectin coacervations by laccase catalyzed cross-linking. LWT-Food Sci. Technol..

[37] Weinbreck F., Nieuwenhuijse H., Robijn G.W., de Kruif C.G. (2003). Complex formation of whey proteins: Exocellular polysaccharide EPS B40. Langmuir.

[38] Azimi S.Z., Hosseini S.S., Khodaiyan F. (2021). Continuous clarification of grape juice using a packed bed bioreactor including pectinase enzyme immobilized on glass beads. Food Biosci..

[39] Ghasemi S., Jafari S.M., Assadpour E., Khomeiri M. (2017). Production of pectin-whey protein nano-complexes as carriers of orange peel oil. Carbohydr. Polym..

[40] Rocha G.A., Fávaro-Trindade C.S., Grosso C.R.F. (2012). Microencapsulation of lycopene by spray drying: Characterization, stability and application of microcapsules. Food Bioprod. Processing.

[41] Einhorn-Stoll U., Archut A., Eichhorn M., Kastner H. (2021). Pectin-Plant protein systems and their application. Food Hydrocoll..

[42] Henry C., Minier J.-P., Pozorski J., Lefèvre G.G. (2013). A new stochastic approach for the simulation of agglomeration between colloidal particles. Langmuir.

[43] Peinado I., Lesmes U., Andrés A., McClements J.D. (2010). Fabrication and morphological characterization of biopolymer particles formed by electrostatic complexation of heat treated lactoferrin and anionic polysaccharides. Langmuir.

[44] Lutz R., Aserin A., Wicker L., Garti N. (2009). Release of electrolytes from W/O/W double emulsions stabilized by a soluble complex of modified pectin and whey protein isolate. Colloids Surf. B Biointerfaces.

[45] Saravanan M., Rao K.P. (2010). Pectin–gelatin and alginate–gelatin complex coacervation for controlled drug delivery: Influence of anionic polysaccharides and drugs being encapsulated on physicochemical properties of microcapsules. Carbohydr. Polym..

[46] Singthong J., Cui S.W., Ningsanond S., Goff H.D. (2004). Structural characterization, degree of esterification and some gelling properties of Krueo Ma Noy (*Cissampelos pareira*) pectin. Carbohydr. Polym..

[47] Assadpour E., Jafari S.-M., Maghsoudlou Y. (2017). Evaluation of folic acid release from spray dried powder particles of pectin-whey protein nano-capsules. Int. J. Biol. Macromol..

[48] Chen Q., Zhong F., Wen J., McGillivray D., Quek S.Y. (2013). Properties and stability of spray-dried and freeze-dried microcapsules co-encapsulated with fish oil, phytosterol esters, and limonene. Dry. Technol..

[49] Moradi S., Khodaiyan F., Razavi S.H. (2020). Green construction of recyclable amino-tannic acid modified magnetic nanoparticles: Application for β-glucosidase immobilization. Int. J. Biol. Macromol..

[50] Zimet P., Rosenberg D., Livney Y.D. (2011). Re-assembled casein micelles and casein nanoparticles as nano-vehicles for ω-3 polyunsaturated fatty acids. Food Hydrocoll..

